# New capabilities of Sentinel-2A/B satellites combined with *in situ* data for monitoring small harmful algal blooms in complex coastal waters

**DOI:** 10.1038/s41598-020-65600-1

**Published:** 2020-05-26

**Authors:** Isabel Caballero, Raúl Fernández, Oscar Moreno Escalante, Luz Mamán, Gabriel Navarro

**Affiliations:** 1 Instituto de Ciencias Marinas de Andalucía (ICMAN), Consejo Superior de Investigaciones Científicas (CSIC), Avenida República Saharaui, 11519 Puerto Real, Spain; 2 Laboratorio de Control de Calidad de los Recursos Pesqueros, Agencia de Gestión Agraria y Pesquera de Andalucía (AGAPA), Consejería de Agricultura, Ganadería, Pesca y Desarrollo Sostenible, Junta de Andalucía, 21459 Cartaya, Spain; 3 Instituto Andaluz de Investigación y Formación Agraria, Pesquera, Alimentaria y de la Producción Ecológica (IFAPA), Centro Agua del Pino, Huelva, 21459 Spain

**Keywords:** Marine biology, Environmental impact, Marine biology, Natural hazards

## Abstract

The increased frequency of harmful algal blooms (HABs) is a major environmental concern worldwide, resulting not only in increased treatment costs for drinking water but also in impacts on tourism, commercial fishing and aquaculture and risks to human and animal health. Traditional strategies with ship-based approaches based on field sampling and laboratory analysis have been adopted to assess HABs. However, these methods are labour intensive and costly and do not provide synoptic views of the bloom conditions. Here, we show that the Sentinel-2 twin satellite mission of the Copernicus programme, in combination with *in situ* data, is a powerful tool that can offer valuable spatiotemporal information about a bloom of the dinoflagellate *Lingulodinium polyedra* that occurred on the SW Iberian Peninsula. Using the robust ACOLITE atmospheric correction processor combined with the normalized difference chlorophyll index (NDCI), the enhanced mapping of small blooms can be performed at a 10 m spatial resolution, revealing surface patches and a heterogeneous distribution. This research also demonstrates the improved capabilities of Sentinel-2 compared to those of Landsat-8 and Sentinel-3 for continuous monitoring. The Sentinel-3 and Sentinel-2 missions provide ecosystem observations that allow the environmental community and water managers to evaluate changes in water quality and bloom distribution and that facilitate field-based measurements. Therefore, the value added by the Copernicus products in terms of frequency and synoptic observations is of paramount importance for ecological and management purposes at regional and national scales.

## Introduction

Phytoplankton blooms are a common occurrence in inland, estuarine and coastal environments worldwide. There are several types of phytoplankton blooms; some are considered significant contributors to ecosystem dynamics and marine primary production, while others are categorized as harmful algal blooms (HABs) or red tides due to their production of potent toxins^1^. Many authors point out that, in term “HAB”, the word “bloom” should be understood with caution, as there are species that show harmful or toxic effects even at low concentration levels^2^. Generally, algal blooms occur when sunlight and high levels of nutrients are available in eutrophic or hypereutrophic waters^2^. HABs are a major global concern that causes hypoxia or anoxia, animal and human health risks, fish deaths, and water and odour problems^3^. The increased duration and frequency of HABs typically during warm periods, has caused high economic costs and severe impacts on commercial fishing, aquaculture, treatment of potable water, tourism, and recreational uses^4^. Ecological and detrimental impacts such as oxygen depletion, habitat alteration, and the displacement of indigenous species can be generated due to phytoplankton blooms that do not produce toxins^5^. HABs are nowadays considered to be one of the most serious coastal environmental concerns, which require further investigation. Additionally, the main conditions leading to the causes of algal blooms and their routine monitoring have still not been fully addressed.

The current strategies used by environmental protection and fisheries agencies include ship-based approaches and *in situ* sampling established on the readily detection of HAB species and toxin levels and periodic coastal monitoring. Generally, monitoring programmes resolve species identification problems, and although there are some species that are more complex to detect, these approaches are an effective strategy for reducing health risk. These standard methods are labour intensive and expensive due to they are based on field sample collection, laboratory analysis, and manual cell counts^6^. In addition, there are still limitations and gaps, mainly associated with the spatiotemporal distribution of blooms, so further research must be carried out for gathering information on their duration and location.

An effective method for monitoring algal blooms and HABs in coastal and offshore waters is to derive chlorophyll-a (chl-a) concentrations as a key indicator of water quality, a proxy for biomass, and a human health guideline for cyanobacteria monitoring^7^. The Water Framework Directive (2000/60/EC) (WFD) obligates all European Union member states to implement water management practices and to estimate the ecological status of their water bodies through monitoring and classification^8^. In this sense, chl-a indicates the trophic status of water and is known as one of the key parameters of the WFD. Hence, frequent mapping in water bodies is essential for fulfilling the WFD. Traditional *in situ* monitoring is a rather time- and money-consuming method for estimating water quality and HABs on a regular basis; however, they are necessary for identifying algae blooms at the species level.

In this context, recurrent and synoptic Earth observation data might offer significant information for improving the monitoring of HABs in coastal ecosystems worldwide. The use of satellite detection techniques based on the optical properties of the water are suitable for filling those gaps and routinely detecting HABs^9,10^. The application of remote sensing is also highlighted by some researchers, who suggested that satellite-derived information is more reliable than traditional monitoring methods for providing information about the distribution of algal blooms^11^. However, the application of remote satellite sensors to measure chl-a, although very important, is not sufficient to detect HABs on its own if we take into account the definition of HABs^12^. Detecting whether a bloom is harmful is not possible only using satellite imagery; additional *in situ* information is required to determine whether the blooms produce toxins. A more holistic and multidisciplinary approach is necessary for optimal risk assessment and can improve our predictive capacity by gathering information from, at a minimum, HAB and biotoxin monitoring data, remote sensors and trajectory modeling. This approach is the starting point for initiatives such as the “Applied simulations and integrated modelling for the understanding of toxic and harmful algal blooms” (ASIMUTH) and “Predicting risk and impact of harmful events on the aquaculture sector” (PRIMROSE) projects^12^.

Red tide or HAB mapping from satellite observations started with the use of chl-a concentration data provided by standard ocean colour sensors, such as the Sea-viewing Wide Field-of-view Sensor (SeaWiFS) and the MODerate resolution Imaging Spectroradiometer (MODIS)^13,14^. Nevertheless, chl-a monitoring over coastal regions has various limitations, such as uncertainties in atmospheric correction, interference from different-coloured compounds, and the presence of suspended material and coloured dissolved organic matter (CDOM), leading to a final overestimation of chl-a^15^. Most methods for HAB detection have been implemented based on SeaWiFS and MODIS because ocean colour sensors have improved signal-to-noise ratio and spectral resolution than terrestrial sensors such as the Landsat-8 (L8) Operational Land Imager (OLI). Regardless of the many benefits of traditional ocean colour sensors, HAB detection often cannot be obtained in complex coastal regions due to coarse spatial resolutions and coastal masking^16^. Because of their moderate spatial resolution, it is extremely challenging to monitor water quality or to detect HABs dynamics within these small aquatic systems. These issues lead to a low quality mapping for HAB patches mixed with saltwater. While numerous studies in Spain have concentrated on the causes of HABs, few have considered the evaluation of new methodologies for the high spatiotemporal mapping of algal blooms. Therefore, there is a pressing need for effective and simple strategies to control the spatiotemporal variability of small coastal algal blooms through remote sensing techniques to assist operational monitoring and emergency plans for the marine environment.

As an alternative to ocean colour sensors, land-focused sensors, which have higher spatial resolution, can be applied. The latest generation of satellites such as the twin Sentinel-2A/B (S2) satellites of the European Union’s Copernicus earth observation programme, carry the MSI (MultiSpectral Instrument) sensor, whose bands have valuable applications for phytoplankton estimation^17–19^ and algal bloom detection^20,21^. The mission was initially designed for the evaluation of urban planning and terrestrial ecosystems, but the incorporation of bands in the red-edge spectrum (704, 740, and 783 nm; see Supplementary Table S1), its radiometric quality (12 bits), its high-revisit frequency (5 days at the equator), and its high spatial resolution (10 m) have proven its utility for coastal and inland waters examination^18,19,22^. In addition, high atmospheric correction accuracy is essential, yet remains a difficult job in turbid and optically complex waters. Considering the need for the fine-scale mapping of algal blooms, the promising capabilities of the S2 spectral features, which are a free public resource, for detecting these phenomena seem to be worth testing.

**Figure 1:**
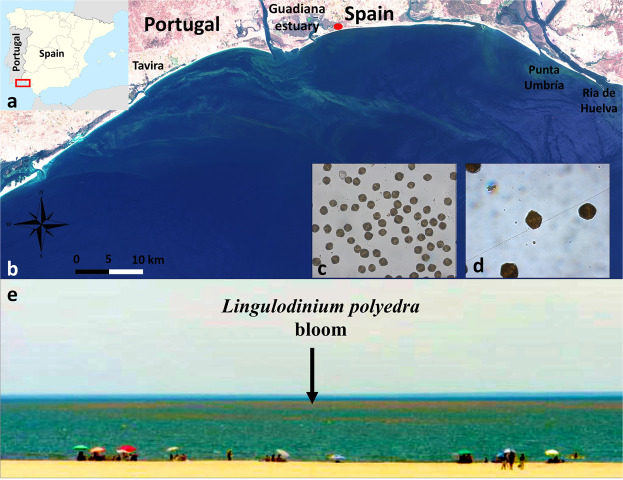
(**a**) Location of the study region in the Guadiana estuary (bordering Spain and Portugal); (**b**) RGB (Bands 4-3-2, Supplementary Table S1) composite from Sentinel-2 captured on 11 July 2019 showing the *L. polyedra* bloom (contains modified Copernicus Sentinel data 2019 processed by Sentinel Hub); (**c**,**d**) *L. polyedra* dinoflagellates identified with a microscope during the bloom; (**e**) photograph of the red bloom in the coastal waters off the Guadiana estuary on 10 July 2019 (photo credit: Manuel Antonio Prado Cruz, Isla Cristina region corresponding to the red dot in Fig. 1b).

A harmful bloom of *Lingulodinium polyedra*, a major HAB species, occurred at the end of June and the beginning of July 2019 in the coastal waters adjacent to the Guadiana estuary (SW Iberian Peninsula, Fig. 1). *L polyedra* is an armoured, marine, bioluminescent dinoflagellate species typically found in neritic waters, which can display phosphorescence at night^23,24^. *L. polyedra* is a widely distributed species that is found in warm-temperate and subtropical waters of coastal areas^25,26^. *L. polyedra* is one of the red tide species known to produce yessotoxins that can accumulate in shellfish with elevated levels of toxicity^27^. Deadly *L. polyedra* HABs have been found from California in the San Diego region^24^ and in the Adriatic Sea, reporting cell levels as high as 2 × 10^7^ cells/liter^28,29^. Sediments from the Guadiana River estuary were analysed for their palynological content, and the analysis revealed the dominance of the autotrophic species *L. polyedra*, which is unusual in stressful environments such as estuaries^30^. However, the levels of yessotoxins in shellfish have never been measured in the Gulf of Cadiz region associated with the presence of this species. In this region, *L. polyedra* is one of the major red tide species responsible for annual blooms at the end of spring or the beginning of summer. Therefore, timely and accurate mapping of widespread HAB distribution in this coastal area is decisive in minimizing the damage and evaluating the environmental impacts of bloom events.

Given this background, the purpose of this study was to analyse the new capabilities of the S2 satellites for estimating the duration, extent and dynamics of an *L. polyedra* bloom in conjunction with *in situ* measurements collected by the relevant regional authority (the Laboratory for the Quality Control of Fishery Resources). A reflectance-based algorithm (normalized difference chlorophyll index, NDCI)^31^ was proposed as an indicator of the dinoflagellate bloom in these turbid coastal waters. We retrieved the spectral reflectance signatures of the algal bloom with the ACOLITE atmospheric correction processor for S2 data as a first attempt to monitor the spatiotemporal distribution of the bloom. We also investigated the specifications of the Sentinel-3 (S3) satellites and analysed the spectral characteristics obtained from S2 and L8 imagery of waters containing *L. polyedra* to determine the advantages and drawbacks of the data from each sensor as support tools for their future incorporation into traditional routine monitoring procedures.

## Results

**Figure 2:**
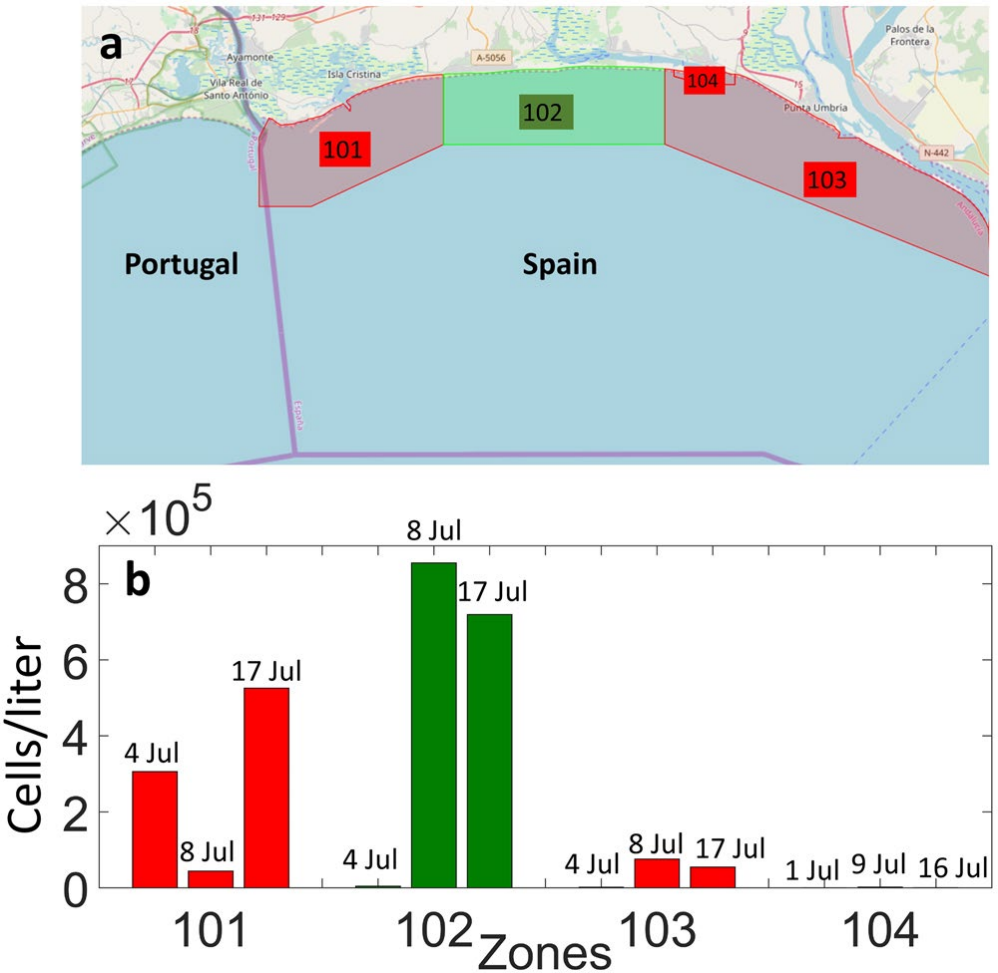
(**a**) Map of the four *in situ* areas (101–104) sampled by the Laboratory for the Quality Control of Fishery Resources; (**b**) estimation of the count of the *L. polyedra* bloom in cells/liter within the four areas from 4 to 17 July 2019.

Based on *in situ* samples from the study area, *L. polyedra* was determined to be the dominant species and the only species with the capacity and sufficient concentration to produce coloration. Red-coloured and green-coloured water was encountered in the coastal region during the bloom period, as shown in the photograph (Fig. 1e). *In situ* water samples showed that red tides did occur in some specific regions, revealing cell counts as high as 8×10^5^ cells per liter in Area 102 (Fig. 2). Area 101 had higher cell count values on 4 and 17 July 2019, whereas Area 102 had a minimum concentration on 4 July 2019 and maximum values on 8 and 17 July 2019, indicating a displacement of the bloom from Area 101 to Area 102. Areas 103 and 104 both showed minimal concentrations during the sampling period, indicating that the bloom extended mainly over Areas 101 and 102. These delimited areas (Fig. 2a) correspond to the typical *in situ* monitoring areas that are routinely and regularly monitored. The samples are not always collected at regular positions, but rather are taken at opportunistic positions. In this specific case, samples were taken in areas showing coloration due to the *L. polyedra* bloom, since the Laboratory for the Quality Control of Fishery Resources specifically aims to assess the intensity of these blooms; the sampling strategy in the official control programme does not establish fixed stations, but it is intended to be representative of the water conditions. Figure 1c,d shows the *L. polyedra* dinoflagellates identified with a microscope during the bloom in the study region.

**Figure 3:**
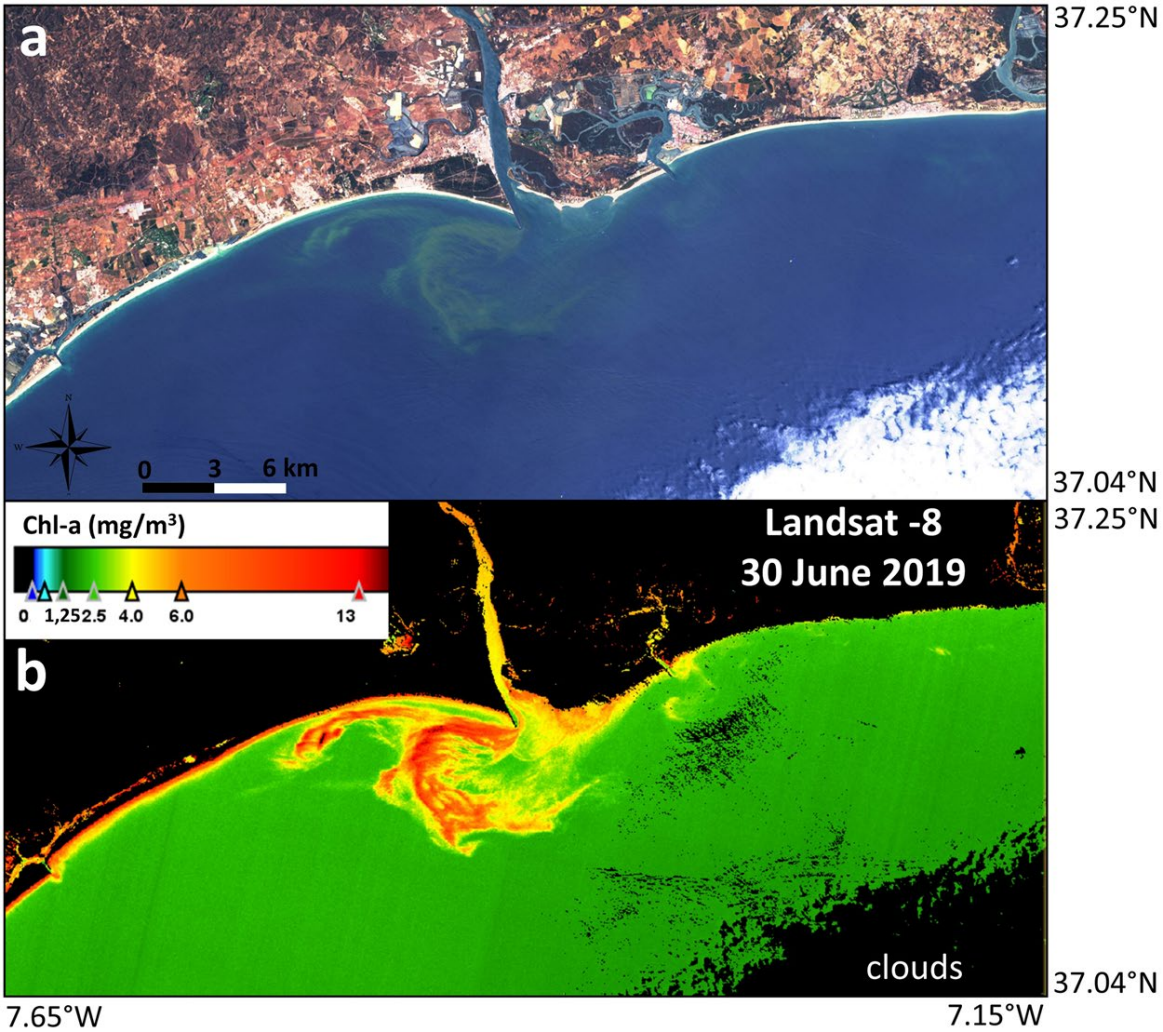
(**a**) Landsat-8 RGB (bands 4-3-2 composite) in the study region on 30 June 2019 showing the *L. polyedra* algal bloom located in the adjacent waters off the Guadiana estuary mouth (contains modified Copernicus Sentinel data 2019 processed by Sentinel Hub); (**b**) chlorophyll-a concentration from the OC3 algorithm (chl-a, mg/m^3^) after atmospheric correction with ACOLITE for the same scene. Maps are generated by SNAP 7.0.0 (https://step.esa.int/main/download/snap-download/). L8 image was downloaded from Earth Explorer (https://earthexplorer.usgs.gov/) and processed with ACOLITE 20190326.0 (https://odnature.naturalsciences.be/remsem/software-and-data/acolite).

On 30 June 2019, the *L. polyedra* bloom was first observed through remote sensing with L8 at 30 m spatial resolution (Fig. 3). Figure 3a shows the coastal region of the Guadiana estuary with an RGB (bands 4-3-2 of L8) composite indicating the location of the bloom confined to an area close to the estuary mouth. The algal bloom can be recognized by a discoloration of the water, manifested as intensely green-coloured water. The NDCI index uses the bands at red 665 nm (Rrs665) and red-edge 708 nm (Rrs708)^31^. Landsat satellites do not have bands within the red-edge spectral region (700–750 nm), so the NDCI cannot be calculated. The distinct optical signature of Sentinel-2/3 compared with that of L8 implies that L8 lacked the necessary optical feature along the red-edge (see Table S1 in Supplementary Information). Therefore, the NDCI was only established for both Sentinel satellites. However, for the L8 scenes, we used the standard OC3 algorithm as a proxy for the quantification of chl-a after the atmospheric correction with the ACOLITE processor. The chl-a map delineated the bloom extension (Fig. 3b). Several days before 30 June 2019 were also evaluated with S2, S3 and L8 data over the region of study, but no bloom was visible.

**Figure 4:**
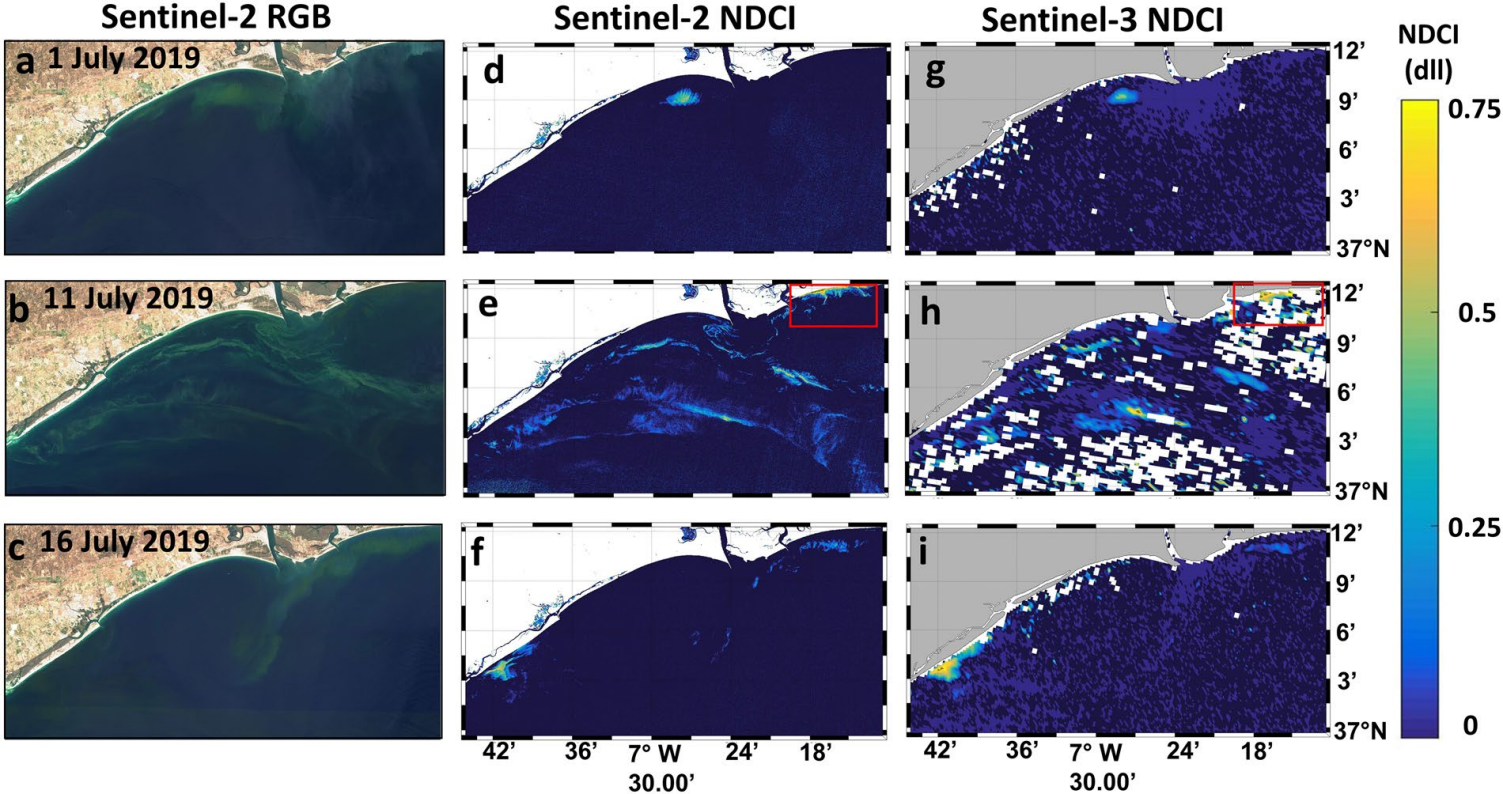
Imagery of the *L. polyedra* algal bloom during the first two weeks of July 2019 in the Guadiana coastal region with Sentinel-2 and Sentinel-3 imagery; (**a-c**) RGB (bands 4-3-2) composite of Sentinel-2 on 1, 11 and 16 July 2019; d-f) NDCI for Sentinel-2 scenes on 1, 11 and 16 July 2019 at 10 m spatial resolution; g-i) NDCI for Sentinel-3 scenes on 1, 11 and 16 July 2019 at 300 m spatial resolution. Contains modified Copernicus Sentinel data 2019 processed by Sentinel Hub. Maps are generated by Matlab R2010b (https://es.mathworks.com/help/matlab/release-notes-R2010b.html). S2 scenes were downloaded from the Sentinel’s Scientific Data Hub (https://scihub.copernicus.eu/) and processed with ACOLITE 20190326.0 (https://odnature.naturalsciences.be/remsem/software-and-data/acolite). S3 scenes were downloaded from the EUMETSAT webpage (https://coda.eumetsat.int).

Figure 4 shows the evolution of the *L. polyedra* algal bloom during the first two weeks of July 2019 (1, 11 and 16 July 2019) in the Guadiana coastal region in true colour and the corresponding NDCI map^31^. Dark blue indicates NDCI values ≤ 0. The most frequently occurring NDCI values varied from 0 to 0.7, implying a moderately high bloom condition^31^. The sun glint was extremely severe in some scenes (4, 6, 9, 14, and 19 July; see Table S2 and Fig. S1 in Supplementary Information), and ACOLITE was not able to perform accurately. These scenes were located in tiles on the eastern side of the swath, so the imagery was extremely sunglint-contaminated. In contrast, for the S2 scenes with tiles located on the western side of the swath (1, 11, and 16 July; see Table S2 and Fig. S1 in Supplementary Information), the sun glint was less intense, and ACOLITE performed accurately.

The RGB (bands 4-3-2) composite of S2 on 1, 11 and 16 July 2019 (Fig. 4a–c, respectively) indicates the location of the bloom each day, as can also be detected with the NDCI generated for S2 (Fig. 4d–f) and S3 (Fig. 4g–i). The true colour composites show a dinoflagellate algal bloom in the coastal waters that gives the water a deep green hue comparable to that of terrestrial vegetation. The algal bloom on 1 July remained close to the estuary mouth (Fig. 4a,d,g). Then, on 11 July, extensive bloom features can be observed in the RGB composite and can also be detected with the NDCI, indicating that the bloom covered the total study region with small patches. On 16 July, the bloom was confined to two regions, one area located east of the estuary mouth and one located southwest of the estuary mouth. These findings were consistent with field observations (Fig. 2) revealing the surface patches of *L. polyedra*.

**Figure 5:**
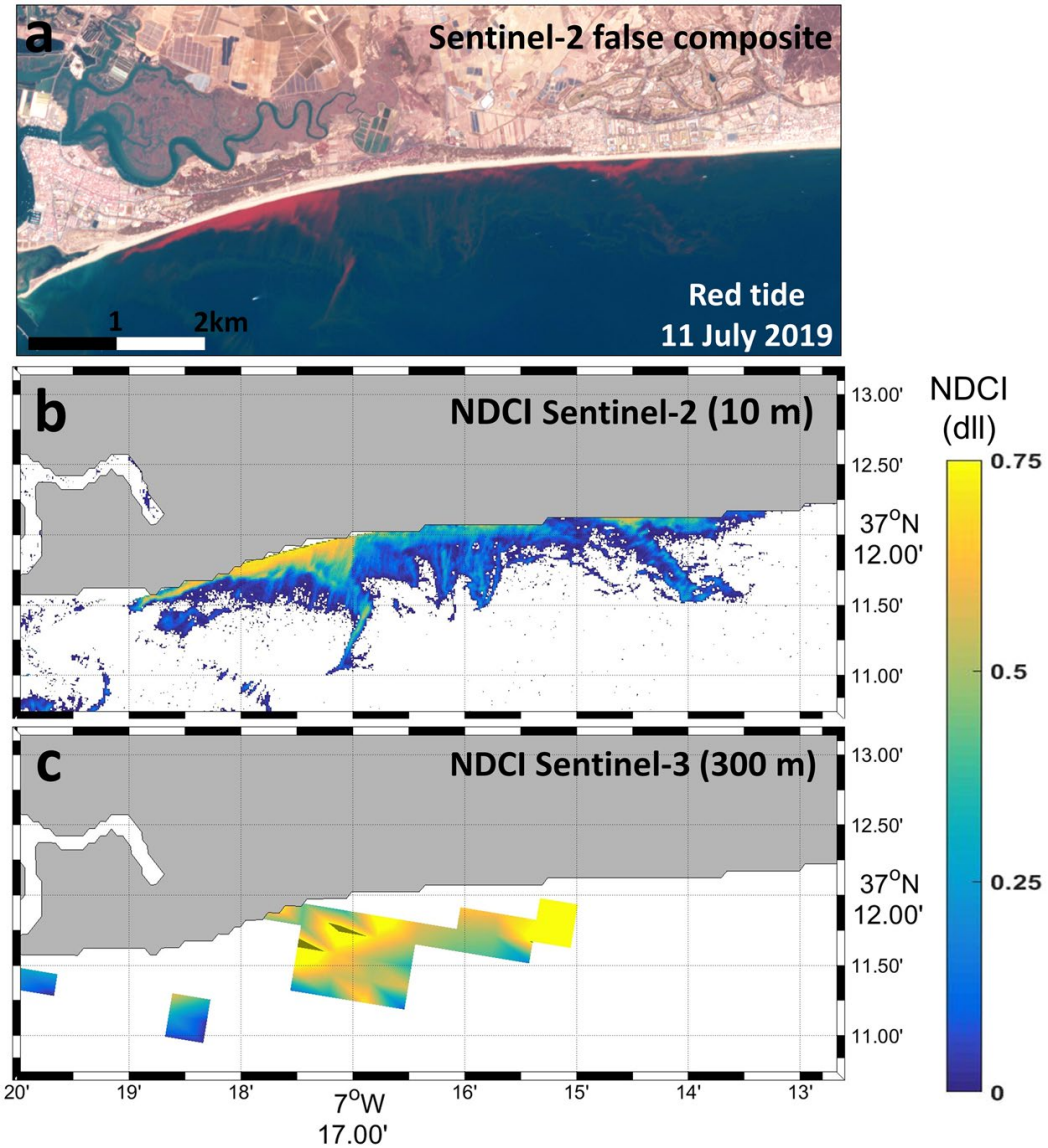
Imagery of the *L. polyedra* algal bloom observed with Sentinel-2 and Sentinel-3 on 11 July 2019. (**a**) False composite (bands 5–3–2) of Sentinel-2 (contains modified Copernicus Sentinel data 2019 processed by Sentinel Hub); (**b**) NDCI (values > 0) for Sentinel-2 at 10 m spatial resolution; (**c**) for Sentinel-3 at 300 m spatial resolution. Maps are generated by Matlab R2010b (https://es.mathworks.com/help/matlab/release-notes-R2010b.html). S2 scene was downloaded from the Sentinel’s Scientific Data Hub (https://scihub.copernicus.eu/) and processed with ACOLITE 20190326.0 (https://odnature.naturalsciences.be/remsem/software-and-data/acolite). S3 scene was downloaded from the EUMETSAT webpage (https://coda.eumetsat.int).

Similar bloom patterns were observed with both Sentinel satellites, noting the different spatial resolutions of S2 at 10 m and S3 at 300 m. Even if S3 is able to detect the bloom, S2 allows the proper visualization and mapping of the small bloom. This is clearly shown by Fig. 5. As a demonstration of the potential of the MSI to forecast the algal bloom distribution at high spatial resolution scales, we derived the NDCI (values higher than zero were masked as an indicator of the algal bloom) in a small area located in the eastern part of the estuary mouth (red rectangle in Fig. 4e,h) on 11 July 2019. The false-colour composite (bands 8–3–2) of S2 (Fig. 5a) depicts a red tide located in the coastal waters close to Isla Cristina (red dot in Fig. 1b), also visible in the photograph taken in front of Isla Cristina on 10 July 2019 (Fig. 1e). S2 is able to properly map the exact extension of the bloom, but S3 did not work accurately. This example is key to showing the potential of S2 to map the bloom at 10 m, in contrast with S3 at 300 m. Many S3 pixels were masked out and excluded due to land/water interference and other artefacts, while S2 offered a complete identification of the bloom. The intense red water colour was caused by the high absorption of blue and green photons and a consequent shift of the reflected light to wavelengths>550 nm^32^. Compared with that of the S3 image, the higher spatial resolution of the MSI image revealed intense small patches of the *L. polyedra* bloom.

**Figure 6:**
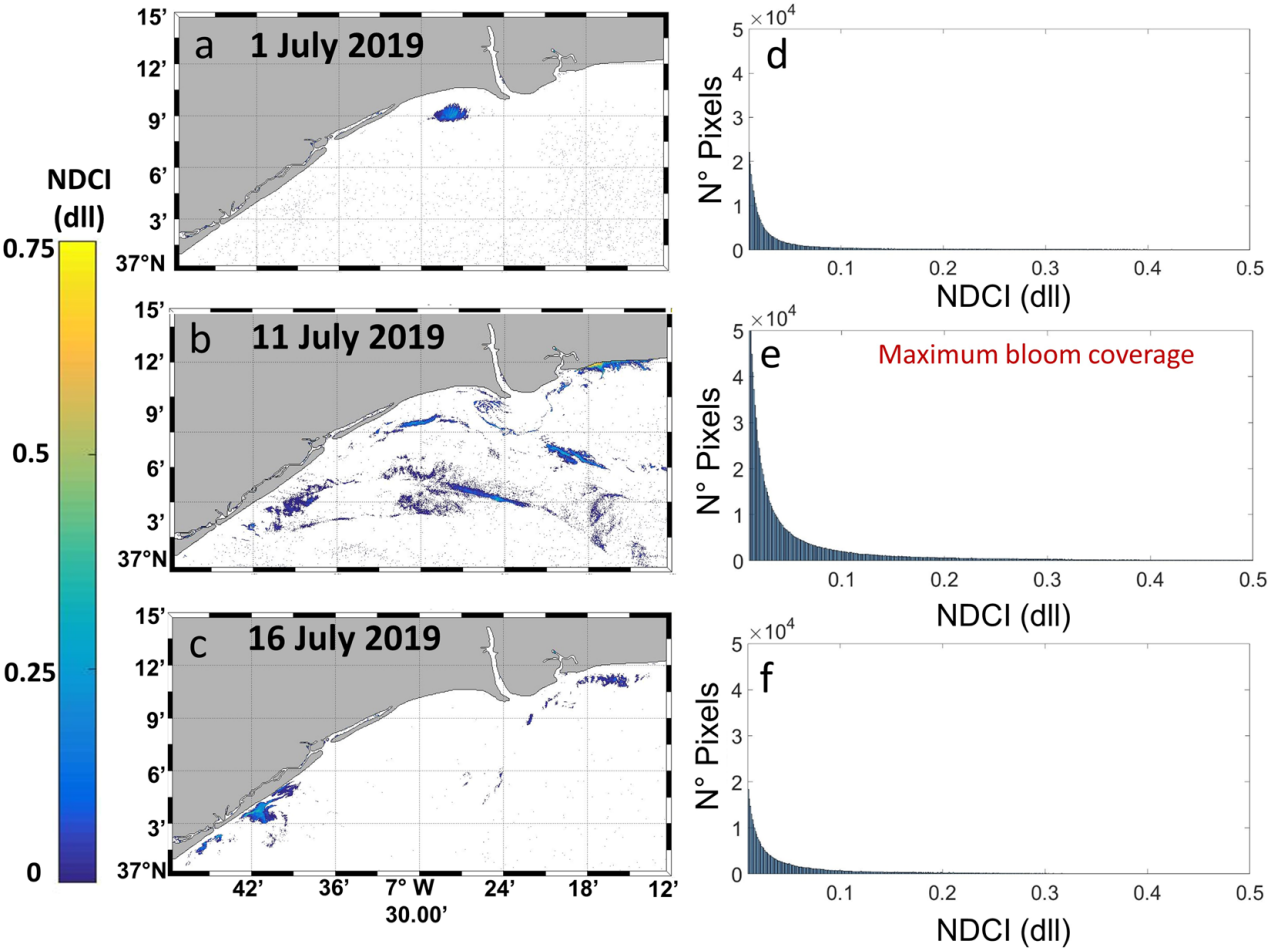
Mask of the *L. polyedra* bloom (NDCI > 0 to 1) for the Sentinel-2 images acquired on (**a**) 1, (**b**) 11, and (**c**) 16 July 2019; (**d–f**) histogram of NDCI for pixels corresponding to the bloom within this region on 1, 11, and 16 July, respectively. Maps are generated by Matlab R2010b (https://es.mathworks.com/help/matlab/release-notes-R2010b.html). S2 scenes were downloaded from the Sentinel’s Scientific Data Hub (https://scihub.copernicus.eu/) and processed with ACOLITE 20190326.0 (https://odnature.naturalsciences.be/remsem/software-and-data/acolite).

The identification of the pixels and their location within the bloom were calculated with the NDCI mask (NDCI > 0 to 1), and the results are shown in Fig. 6a for 1 July, Fig. 6b for 11 July, and Fig. 6c for 16 July 2019. The maximum bloom coverage was noted for the scene of 11 July, where the patchiness of the bloom was spread over the region of the study from coastal waters to offshore areas. The histograms of NDCI for each day also indicated 11 July as the maximum number of pixels, highlighting a peak of the bloom as opposed to the confined bloom areas on 30 June (Fig. 3) and 1 July. (Fig. 4). These bloom patterns are in accordance with the *in situ* observations (Fig. 2).

**Figure 7:**
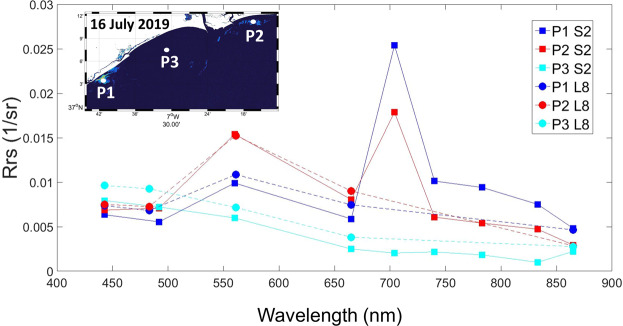
Example of the spectral features from Sentinel-2 (S2, solid lines with squares) and Landsat-8 (L8, dashed lines with circles) at the three control points in the NDCI image on 16 July 2019: P1 (NDCI = 0.62) and P2 (NDCI = 0.38) within the *L. polyedra* bloom and P3 (NDCI = −0.1) outside the bloom. Maps are generated by Matlab R2010b (https://es.mathworks.com/help/matlab/release-notes-R2010b.html).

To evaluate the spectral capabilities of S2 compared to those of L8 for algal bloom mapping, Fig. 7 shows the spectral signal obtained after atmospheric and sun glint correction with ACOLITE DSF on 16 July 2019 (the only coincident date for both S2 and L8). Three control points were selected based on the NDCI values, and two of them were within the algal bloom: P1 was located southwest of the Guadiana estuary mouth (NDCI = 0.62), P2 was located in the eastern part of the estuary (NDCI = 0.38), and P3 was located outside the bloom (as indicated by the negative values of NDCI = −0.1). First, it is worth mentioning that the Level 2 bottom of atmosphere (BOA) reflectances indicated that the atmospheric and sun glint corrections performed similarly for both satellites. ACOLITE provided corrected visible bands for both L8 and S2 at the three control points. This can be seen in P3, where the spectral signature is comparable along the visible and NIR spectra. However, the additional red-edge bands of S2 (704, 740, and 783 nm; see Table S1 in Supplementary Information) were able to provide information about the bloom, specifically the 704 nm band (used for NDCI). The peak at 704 nm noted at both P3 and P1 (the P1 peak was more acute due to its higher NDCI levels) was a clue to masking the algal bloom extension by means of the NDCI. The spectral characteristics of L8 are limited within this red-edge part of the spectrum.

## Discussion

**Figure 8:**
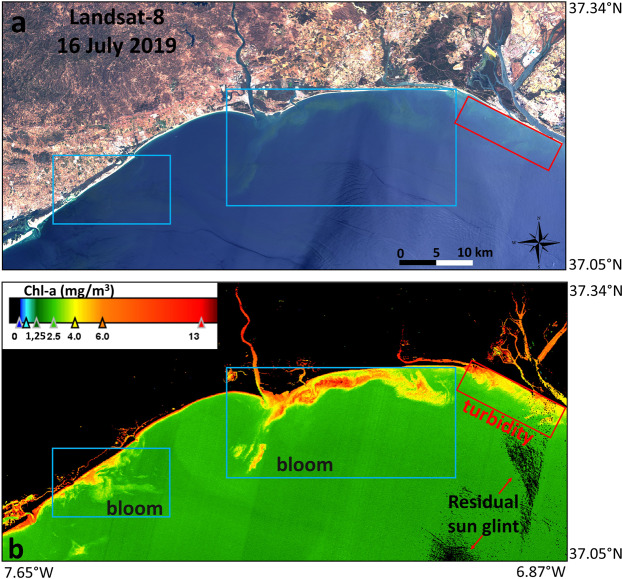
(**a**) Landsat-8 RGB (bands 4-3-2) composite in the study region on 16 July 2019 showing the *L. polyedra* algal bloom located in the waters off the Guadiana estuary mouth (contains modified Copernicus Sentinel data 2019 processed by Sentinel Hub); (**b**) chl-a concentration (mg/m^3^) after atmospheric correction with ACOLITE for the same scene. Some residual sun glint after atmospheric correction is visible in the lower right panel of the chl-a map (black pixels). Blue rectangles indicate high concentrations of chl-a due to the algal bloom, whereas the red rectangle indicates a high chl-a concentration due to high turbidity in the Tinto-Odiel estuary (Ria de Huelva and Punta Umbria, Fig. 1b). Maps are generated by SNAP 7.0.0 (https://step.esa.int/main/download/snap-download/). L8 image was downloaded from Earth Explorer (https://earthexplorer.usgs.gov/) and processed with ACOLITE 20190326.0 (https://odnature.naturalsciences.be/remsem/software-and-data/acolite).

An encouraging method for routinely monitoring algal blooms and for HAB detection in coastal and inland waters is to use both space-based platforms with high spatial, temporal and spectral resolution and *in situ* sampling^33^. Detecting whether a bloom is harmful is not possible only using satellite imagery, so additional *in situ* information is required to determine whether the blooms produce toxins. For an optimal risk assessment and response to such a natural disaster, a multidisciplinary approach is recommended that includes gathering information from HAB monitoring data, remote sensors and modelling. In ocean colour remote sensing applications, the atmospheric correction model might perform accurately since any error from the atmospheric correction stage might affect the estimation of the biophysical parameters^31^. S2 is mainly designed for vegetation applications, so a precise atmospheric correction scheme is required for the development of water quality algorithms. In this study, some S2 images acquired on 4, 6, 9, 14, and 19 July 2019 were of poor quality after ACOLITE due to extremely severe sun glint effects, so the affected pixels were flagged and masked out. The reason for the extreme sun glint was that these tiles were located on the eastern side of the swath. In contrast, for the S2 scenes with tiles located on the western side of swath (1, 11, and 16 July 2019), the sun glint was less intense, and ACOLITE performed accurately. Some examples of the RGB composites at the TOA and BOA levels (before and after the ACOLITE atmospheric correction, respectively) are presented in Figure S1 (Supplementary Information) for S2 scenes acquired in July 2019. The extreme sun glint effects are clearly visible at both TOA and BOA on 4 July 2019 due to the eastern tile position (Fig. S1c,d) compared to the scene on 1 July 2019 (Fig. S1a,b). While the bloom is clearly visible at the BOA level at the mouth of the estuary on 1 July, on 4 July, the bloom is not well distinguished at the mouth due to residual sun glint. In this regard, working in this region during summer might require S2 scenes/tiles located on the western side of swath to address the loss of data due to sun glint. In addition, if sun glint issues during summer may be even more prevalent in other regions due to the orbit characteristics of the satellites, further atmospheric correction processors (e.g., C2RCC in the SNAP platform) might also be tested. For the L8 scenes, ACOLITE was able to perform accurately, and minimal sun glint effects are visible in the images. One example of the residual sun glint after atmospheric correction is visible in the lower right panel of the chl-a map (Fig. 8). Nevertheless, ACOLITE seemed to perform correctly for both satellites and provided similar Rrs values for pixels within and outside the algal bloom (Fig. 7). Recent works have already suggested that ACOLITE offers accurate information for water quality monitoring in complex waters in Spain, such as in the Guadalquivir estuary^22^, located southeast of the Guadiana estuary, and in the largest coastal lagoon in the Mediterranean^34^. A more exhaustive evaluation of the ACOLITE method, usually with *in situ* radiance measurements, needs to be considered for a more comprehensive comparison of processor performance for coastal water quality studies in this region^35,36^.

The accurate retrieval of biophysical parameters such as phytoplankton, chl-a, and algal bloom extent from remote sensing in turbid coastal productive waters is crucial for multitemporal and large-scale evaluations associated with biogeochemical cycles, marine primary production, and inland and coastal water quality. Empirical standard algorithms (e.g., OC3) are frequently applied for chl-a mapping and offer accurate estimation in clear waters. In contrast, these algorithms do not provide reasonable accuracy in optically complex waters such as inland and coastal regions were non-covarying, optically active constituents such as CDOM and suspended matter are present^15^. Figure 8 shows an example of this factor in a chl-a map from L8 on 16 July 2019. The *L. polyedra* algal bloom is located in the waters adjacent to the Guadiana estuary, and the blue rectangles indicate high chl-a concentrations due to the algal bloom. The red rectangle also indicates high chl-a due to turbidity in the Tinto-Odiel estuary (Ría de Huelva, Fig. 1), thus overestimating the chl-a concentration. It should be noted that this region is relatively influenced by coastal and riverine water constituents such as suspended sediments^37–40^.

Phytoplankton productivity and algal blooms in coastal, estuarine, and inland water environments are frequently inferred from the shape of the visible and near-infrared spectra obtained from remote sensing observations. The spectral curves in this study show that the pixels within the algal bloom often exhibited acute reflectance peaks in the MSI red-edge band (704 nm), whereas minimum values were encountered for non-bloom pixels (Fig. 7). Similar reflective spectra in the red-edge bands have also been observed by several researchers and have usually been associated with algal blooms^18,19,41–43^. The prominent reflectance peak located in the 700–720 nm region shifts towards longer wavelengths as phytoplankton concentrations increase; thus, the 704 nm band is suggested as the best option for HABs mapping^19^. Sakuno *et al*. already indicated the potential of the 704 nm red-edge band of S2 for red tide monitoring in Japan^21^. The sensitivity of NDCI to chl-a concentration in turbid productive waters has been demonstrated, as well as its potential application to several platforms and diverse environments producing minimal uncertainty^31^. As the red-edge bands of S2 and S3 (704 nm and 708 nm, respectively, Supplementary Table S1) are required for the calculation of NDCI, both missions allow the monitoring of algal blooms in optically complex and productive estuarine and coastal waters such as those in this coastal study region^37–40^.

Landsat satellites do not have bands in the red-edge spectral range (700–720 nm). The above-mentioned distinct optical signature of the Sentinels compared to that of L8 suggests that L8 lacks the necessary optical feature in the red-edge bands (Fig. 7). Additionally, one of the benefits of the straightforward relationship between the NDCI values and the chl-a concentrations is the intention to make NDCI simple and appropriate when/where ground-truthed data are not available, as in this case study; this feature makes NDCI extensively applicable to estuarine and coastal waters, as NDVI is to terrestrial vegetation^31^. A synoptic scene can provide significant information about the location and distribution of HABs as well as bloom detection using the NDCI. The patchy nature of the bloom and its irregular patterning were reported at the sea surface during typical relaxed summer conditions. These findings demonstrated that NDCI is effective for monitoring HABs with S3 and S2 data and verification using *in situ* data (Fig. 2). For the first time, to our knowledge, the *L. polyedra* bloom dynamics was monitored in this region based on the spectral signature at the red-edge bands of S2 and S3. Recent studies have shown the potential of NDCI for mapping algal blooms and chl-a concentrations^18,44–46^.

Although monitoring the spatiotemporal evolution of the harmful *L. polyedra* algal bloom was possible using S2 and S3 (Fig. 4), the small size and the patchiness of the algal bloom (Fig. 5a) limited its detection with S3 (Fig. 5c). This species usually forms very elongated and thin slicks with widths varying from a few to tens of metres, so OCLI’s spatial resolution (300 m) is not sufficient to resolve the patchiness. In contrast, S2 was able to accurately detect and map the bloom extension close to the coast thanks to its 10 m spatial resolution (Fig. 5b). The small pixel size of the S2 mission is a main advantage in the remote sensing of small algal blooms compared to that of S3. The NDCI algorithm for both S2 and S3 can provide reliable and relevant information in quasi-real time to prepare for and respond to red tides as well as for determining the bloom distribution during the HAB event. In addition, the S2 imagery permits the detection of bloom patches of smaller extents and in earlier stages, which might help to alert coastal managers about the potential impact of the red tides in coastal regions. This simple algal bloom mapping strategy can be used in combination with standard *in situ* observations to control and manage HABs in small reservoirs, and inland waters and bloom areas can be quickly and accurately estimated due to their fine-scale resolution (Fig. 6).

Currently, both S2A and S2B are in orbit, providing a five-day revisit time at the equator and better temporal resolution at higher latitudes. This will allow continuous monitoring for algal blooms in small geographic areas where the 300 m spatial resolution of S3 is not sufficient. In addition, a multisensor approach using both S2 and S3 is also suitable for the operational and complete monitoring of algal bloom detection over broader regions. These new capabilities offer coastal water managers novel powerful tools for assessing the condition of their water bodies more frequently and synoptically, which will allow them to focus their limited resources on mitigating the risks of potentially harmful blooms at regional to national scales. The advantage of using next-generation optical sensors such as S2 to supplement the information gathered from *in situ* observations of algal bloom dynamics is of key importance^47,48^. Sediments from the Guadiana River estuary were analysed for their palynological content, and the dominance of the autotrophic species *L. polyedra* was revealed, which is unusual in high-stress environments such as estuaries^30^. In their study, the high concentration and clear dominance of the species in the top layer of the estuary sediments probably reflected the occurrence of a large bloom of this yessotoxin-producing species in the adjacent coastal waters and their subsequent transport and sedimentation into the estuary. It is suggested in that study that intensive monitoring of phytoplankton and bloom compositions should be performed in the Guadiana estuary and the adjacent coastal zone. Considering that the S2 mission will provide images over the coming decades, this approach could be applied for the effective routine monitoring of algal blooms along this coast, aiding in the routine mapping requirements of the EU WFD. Further research should be focused on gathering field data covering a higher number of algal bloom scenarios; however, the results of this study indicate the potential of S2 as a tool for assessing the spatiotemporal dynamics of algal blooms through holistic (time-series) analysis and for encouraging improved coastal management practices for the Guadiana estuary and its adjacent coastal waters in the future.

## Conclusions

The S2A/B twin satellites were not primarily designed to observe the optical properties of water; however, this study proves that they have suitable capabilities for supporting the environmental monitoring of the small HAB bloom that occurred during summer 2019 in the coastal waters adjacent to the Guadiana estuary (SW Iberian Peninsula). *In situ* water sampling revealed that the dinoflagellate species was *Lingulodinium polyedra*. The obtained results confirm the robustness of the ACOLITE atmospheric correction model combined with NDCI for mapping the bloom extension using S2 imagery. We suggest the usage of NDCI as a simple and feasible model that can be applied to multi-sensor ocean colour data, such as those from Sentinel-2 and Sentinel-3, for algal bloom detection in turbid complex coastal waters. The red-edge bands on S2 (704 nm) and S3 (708 nm) allowed for the calculation of NDCI and the subsequent comprehensive mapping of the algal bloom at unprecedented spatial scales in highly productive near-shore coastal waters. The extent and duration of the bloom could be deduced from remote sensing; consistent with field observations, the remote imagery indicated surface patches of *L. polyedra*. S3 has superior temporal and spectral resolutions to MSI; however, it has a limited spatial resolution, complicating the detection of small algal blooms in coastal areas with complex coastlines. This research demonstrated the strong advantage of S2 (10 m) over S3 (300 m) data for the analysis of small blooms, as well as the better spectral, spatial and temporal resolution of S2 than of L8 for continuous monitoring. The timely and accurate mapping of the widespread HAB distribution in this coastal region is crucial for minimizing the damage and evaluating the environmental impacts of the annual bloom events. Therefore, the value added by the Copernicus products in terms of their frequency and synoptic observations is of paramount importance for water quality monitoring plans and for ecological and management purposes at regional and national scales.

## Methods

### **Study region and*in situ*data**

The Guadiana River basin (on the southwestern Iberian Peninsula) originates in Spain and flows south to the Atlantic Ocean through Portugal (Fig. 1). The Guadiana estuary, bordering both Spain and Portugal, is a mesotidal estuarine system located in a temperate climate area, with moderate, humid winters and hot, dry summers^49^. In the lower estuary, the valley opens into a coastal plain, with a large round marsh areaand barrier islands at the seaward end^50^. The freshwater inputs to the estuarine zone vary sharply, depending on the rainfall and on water retention in upstream dams^51^. A total of 2 million people inhabit the Guadiana River basin, ~90% of whom are in Spain. The extensive development of the estuary over the last century has resulted in significant alteration of river flow regimes as well as anthropogenic nutrient enrichment^52^. In the last 20 years, water-development projects have been constructed in this basin to supply water for agriculture and human consumption^53^. Damming, water abstraction, and agricultural, industrial, touristic, and urban pressures are threats to the lower Guadiana estuary^51^.

The study area is part of Andalusian community and, therefore, is subject to official controls of toxic phytoplankton and biotoxins to comply with European regulations (EC 853/2004 and EC 854/2004). The strategy is based on weekly sampling, except in the case of toxicity intensification of molluscs and the water column in each production area as defined by the authority for the area (the Laboratory for the Quality Control of Fishery Resources) in the Order of 15 July 1993, Boja No. 85 (5 August 1993), updated on successive occasions. The sampling point within each zone is usually based on the locations of the greatest fishing activity and on the location of oceanographic data collection stations. Water samples are collected so that they integrate the entire water column through a system of interconnected hoses. In addition, a concentrated sample was obtained by means of vertical dragging (bongo-type net to a size of 20 µm) to allow the detection of species at a very low concentration. The analysis of the samples was carried out by taking an aliquot of sediments and examining the material in an inverted Utermöhl microscope, as recommended by the European UNE-EN 15204. The laboratory quality system has been accredited by the national entity ENAC since 2007 (UNE-EN-ISO 17025). The toxic phytoplankton identification and counting techniques were added to those accredited in the laboratory in 2011. Figure 2a shows a map of the four *in situ* areas (101–104) sampled for the count of the *L. polyedra* bloom (cells/liter) within the productivity zones in the Guadiana estuary and the adjacent region from 4 to 17 July 2019 (http://www.juntadeandalucia.es/agriculturaypesca/moluzonasprodu/). More detailed information on the sampling methods can be obtained in this work^6^. The *in situ* sampling areas (Fig. 2a) are routinely and regularly monitored. The samples are not always collected at regular positions, but at opportunistic positions. In this specific case, samples were taken at areas showing coloration due to the *L. polyedra* bloom, since the Laboratory for the Quality Control of Fishery Resources precisely aims to assess the intensity of these blooms. The sampling strategy of the official control programme does not establish fixed stations, but it is intended to be representative of the water body.

### Satellite data

#### Sentinel-3 and Sentinel-2

The European Space Agency (ESA) in collaboration with the European Commission (EC) developed the Sentinel fleet to meet the operational needs of the Copernicus programme. Each Sentinel mission uses a constellation of two satellites to fulfil revisit and coverage requirements and thereby provides robust data sets for Earth observation services. In this study, S2 and S3 satellites were used; data from both constellations are available openly and freely for all users.

S3 is a multisensor mission comprising two satellites (S3A and S3B) in identical orbit with a phase shift of approximately 140°. S3A has been in orbit since February 2016, and S3B has been in orbit since 25 April 2017; both satellites were operational at the time of the bloom. The Ocean and Land Colour Instrument (OLCI) onboard S3 is a medium-resolution imaging spectrometer that uses five cameras to provide a wide field of view (swath width: 1270 km) with 14-bit radiometric resolution and enhanced long-term radiometric stability^54^. It provides 21 bands, ranging from the visible to the near infrared (400–1020 nm, Supplementary Table S1), acquired simultaneously with 300 m spatial resolution. The revisit frequency is 1–2 days. The main objective of this mission is to accurately measure sea-surface topography, sea- and land-surface temperatures and ocean- and land-surface colour in support of ocean forecasting systems and environmental and climate monitoring. Standard OLCI Level-2 Water Full Resolution (OL_2_WFR) products were downloaded from the EUMETSAT webpage (https://coda.eumetsat.int). OLCI images coinciding with S2 scenes in the study region from 1 to 20 July were selected for analysis (Supplementary Table S2). Remote sensing reflectance corrected for the atmosphere and for sun specular reflection, hereinafter referred to as Rrs (1/sr), in all visible and NIR bands was used, as well as chl-a concentration data computed using “OC4Me” or neural network algorithms^54^.

The S2 twin-satellite mission has established a new era in coastal monitoring due to its acquisition repeat frequency of 5 days, high spatial resolution of 10–20–60 m (see Supplementary Table S1 for detailed information on spectral bands), and more significantly, its free and open data access policy^55^. The S2 mission is based on a constellation of two identical satellites in the same orbit, phased at 180° to each other. Both satellites, −2A (launched on 23 June 2015) and −2B (launched on 7 March 2017), provide optimal coverage of Earth’s land surfaces, large islands and inland and coastal waters. While the mission was designed primarily for land applications, their imaging sensor characteristics match those proposed by the remote sensing community for effective and accurate coastal and inland water monitoring^56^. Owing to the technical capabilities offered by the S2 fleet, scientists have begun to take into consideration this mission as a suitable solution for coastal research mapping in Spain^22,34^. The radiometric resolution of MSI is 12 bits, and the spectral characteristics of the bands used in this study are shown in Supplementary Tables S1 and S2 scenes for the study area were downloaded from the Sentinel’s Scientific Data Hub (https://scihub.copernicus.eu/). These images corresponded to Level-1C (L1C) radiometrically and geometrically corrected Top Of Atmosphere (TOA) products^55^. The calibrated TOA reflectance products are provided to the science/user community at their native spatial resolution in 100 km × 100 km tile formats^55^. In this study, the images of zone 19 (subtile SPB) were used. Only scenes with low cloud coverage over the study region were selected for further analysis, with a total of 8 suitable scenes from 1 to 19 July (see Supplementary Table S2 for detailed information on the S2A/B scenes used).

#### Landsat-8

Landsat-8 (L8), a Department of the Interior U.S. Geological Survey (USGS) and National Aeronautics and Space Administration (NASA) collaboration, carries two sensors, the Operational Land Imager (OLI) and the Thermal Infrared Sensor. L8 acquires imagery of the Earth’s terrestrial and polar regions at a moderate spatial resolution in the visible, near-infrared, short wave, and thermal infrared spectra. L8 scenes complement the historical observations acquired by previous Landsat missions from the U.S. Landsat archive, which are freely available^57^. Compared to Landsat heritage sensors, the OLI onboard L8 has improved characteristics, which include an improved signal-to-noise ratio and 12-bit radiometric resolution. The OLI spectral bands remain roughly comparable to the Landsat 7 Enhanced Thematic Mapper plus (ETM+) bands, including two additional bands: a new infrared band (1.36–1.39 μm) for cirrus cloud detection and a new shorter blue band (0.43–0.45 μm) intended to retrieve atmospheric aerosol properties and to provide enhanced sensitivity to chlorophyll and other suspended materials in coastal and inland waters^57^. The satellite has a 16-day repeat cycle with an equatorial crossing time of 10:00 a.m. Since 2013, the advanced data quality of OLI has expanded the existing applications of Landsat imagery in aquatic sciences based on the retrieval of near-surface parameters at 30 m resolution^58^. Orthorectified and terrain corrected Level 1 T OLI images were downloaded free of charge from Earth Explorer (https://earthexplorer.usgs.gov/). The tiles corresponding to the region of interest were in row 202 (034), and 3 images were selected for processing (Supplementary Table S2), with a scene size of 170 km × 185 km.

## Atmospheric Correction

All the L8 and S2 Level 1 images were atmospherically corrected to the BOA level. Currently, several open-source atmospheric correction algorithms are available for both satellite missions. In this study, images were processed to Level-2A (L2A) with one of the most common software programs used, the ACOLITE processor 20190326.0 (https://odnature.naturalsciences.be/remsem/software-and-data/acolite). ACOLITE is an image-based approach that does not require *in situ* atmospheric information. ACOLITE was specifically developed for marine, coastal and inland waters by the Royal Belgian Institute of Natural Sciences (RBINS) and supports free processing of both L8 and S2^17^. We selected a novel algorithm within the ACOLITE toolbox, the Dark Spectrum Fitting (DSF) atmospheric correction model^59^. This approach addresses some of the common problems of the exponential model with a robust automated band selection process and an aerosol correction; these processes account for the spatial variability of aerosols (in both type and concentration) without affecting the noise level in the output product^59,60^. This recent algorithm was initially developed for water applications of metre-scale optical satellites but has already indicated its potential for application to S2^60^ due to their improved spectral coverage (notably including bands in the SWIR region). In this study, we also selected this algorithm with the optional image-based sun glint correction of the surface reflectance. ACOLITE products correspond to remote sensing reflectance (Rrs, 1/sr) in all visible and NIR bands, resampled to 10 m pixel size for S2 and 30 m for L8. For L8, the chlorophyll-a concentration (chl-a) was also generated using the OC3 algorithm with ACOLITE as a proxy of the bloom extent.

## Normalized Difference Chlorophyll Index (NDCI)

The normalized difference chlorophyll index (NDCI, Eq. 1), a band-difference algorithm, was used to accurately map the bloom area from remote sensing data in estuarine and coastal turbid productive waters^31^. Findings from that research suggest that NDCI results derived from simulated and Medium Resolution Imaging Spectrometer (MERIS) datasets show its potential application to widely varying water types and geographic regions^31^. In the case of remote coastal waters with no chl-a ground-truthed data, such as this case study, NDCI can be used to detect algal blooms and qualitatively infer chl-a concentration ranges; this process is very similar to NDVI’s application in terrestrial vegetation studies. NDCI uses the bands at 665 nm (Rrs665) and 708 nm (Rrs708), emulating the MERIS channels. The two spectral features centred at the red 665 nm and red-edge 708 nm were selected to develop NDCI (dimensionless) and to avoid the confounding influence of CDOM and total suspended solids (TSS) on the water reflectance spectra at shorter wavelengths. Both S3 (band 8 and band 11) and S2 (band 4 and band 5) have the specific bands for determining the NDCI, whereas L8 only has the red band. Therefore, the NDCI was only established for both Sentinel satellites. Following the recommendation of a previous study, NDCI values higher than zero were masked as an indicator of the *L. polyedra* algal bloom in the Guadiana-adjacent coastal waters^31^.

1 $${\rm{NDCI}}=\frac{Rrs708-Rrs665}{Rrs708+Rrs665}({\rm{dll}})$$

## Supplementary information

Supplementary Information.

## Data Availability

The dataset generated and analysed during the current study is available from the corresponding author upon request.
