# Supplementary material for: New capabilities of Sentinel-2A/B satellites combined with in situ data for monitoring small harmful algal blooms in complex coastal waters
Source: Sci Rep. 2020 May 26;10:8743. doi: 10.1038/s41598-020-65600-1 (PMC7250863; doi:10.1038/s41598-020-65600-1)
Supplement: Supplementary file 1 — Supplementary Information. [file 41598_2020_65600_MOESM1_ESM.pdf]

New capabilities of Sentinel-2A/B satellites combined with *in situ* data for monitoring small harmful algal blooms in complex coastal waters

Isabel Caballero[1*], Raúl Fernández[2], Oscar Moreno Escalante[3], Luz Mamán[2], and Gabriel Navarro[1]

1. Instituto de Ciencias Marinas de Andalucía (ICMAN), Consejo Superior de Investigaciones Científicas (CSIC), Avenida República Saharaui, 11519, Puerto Real, Spain.
2. Laboratorio de Control de Calidad de los Recursos Pesqueros, Agencia de Gestión Agraria y Pesquera de Andalucía (AGAPA), Consejería de Agricultura, Ganadería, Pesca y Desarrollo Sostenible, Junta de Andalucía, 21459, Cartaya, Spain.
3. Instituto Andaluz de Investigación y Formación Agraria, Pesquera, Alimentaria y de la Producción Ecológica (IFAPA), Centro Agua del Pino, 21459, Huelva, Spain

* Correspondence: isabel.caballero@icman.csic.es

Table S1. Specifications of the satellite data used in this study: Sentinel-3 (300 m, daily revisit), Sentinel-2 (10-20-60 m, 5-day revisit), and Landsat-8 (15-30 m, 16-day revisit).

**Sentinel-3**		**Sentinel-2**		**Landsat-8**	
Band	Wavelength (nm)	Band	Wavelength (nm) / spatial resolution (m)	Band	Wavelength (nm) / spatial resolution (m)
1	400	1	443 / 60	1	443 / 30
2	412.5	2	490 / 10	2	483 / 30
3	442.5	3	560 / 10	3	561 / 30
4	490	4	665 / 10	4	655 / 30
5	510	5	704 / 20	5	865 / 30
6	560	6	740 / 20	8	590 / 15
7	620	7	783 / 20		
8	665	8	842 / 10		
9	673	8a	865 / 20		
10	681				
11	708				
12	753				
13	761				
14	764				
15	767				
16	778				
17	865				
18	885				
19	900				

Table S2. List of imagery from Sentinel-3 (S3), Sentinel-2 (S2), and Landsat-8 (L8) for the study period during summer 2019. Intense sun glint did not allow retrieving any information with the ACOLITE processor for S2 tiles located on the eastern side of the swath (4, 6, 9, 14, and 19 July 2019). Conversely, for the S2 scenes with tiles located on the western side of swath (1, 11, and 16 July 2019), sun glint was less severe, and ACOLITE performed accurately.

Satellite	Sensing date	Observations
L8	20190630	Bloom
S2A	20190701	Bloom
S3B	20190701	Bloom
S2A	20190704	Intense sunglint
S2B	20190706	Intense sunglint
S3A	20190706	Bloom
S2B	20190709	Intense sunglint
L8	20190709	Bloom
S2A	20190711	Bloom
S3A	20190711	Bloom
S2A	20190714	Intense sunglint
S2B	20190716	Bloom
S3B	20190716	Bloom
L8	20190716	Bloom
S2B	20190719	Intense sunglint

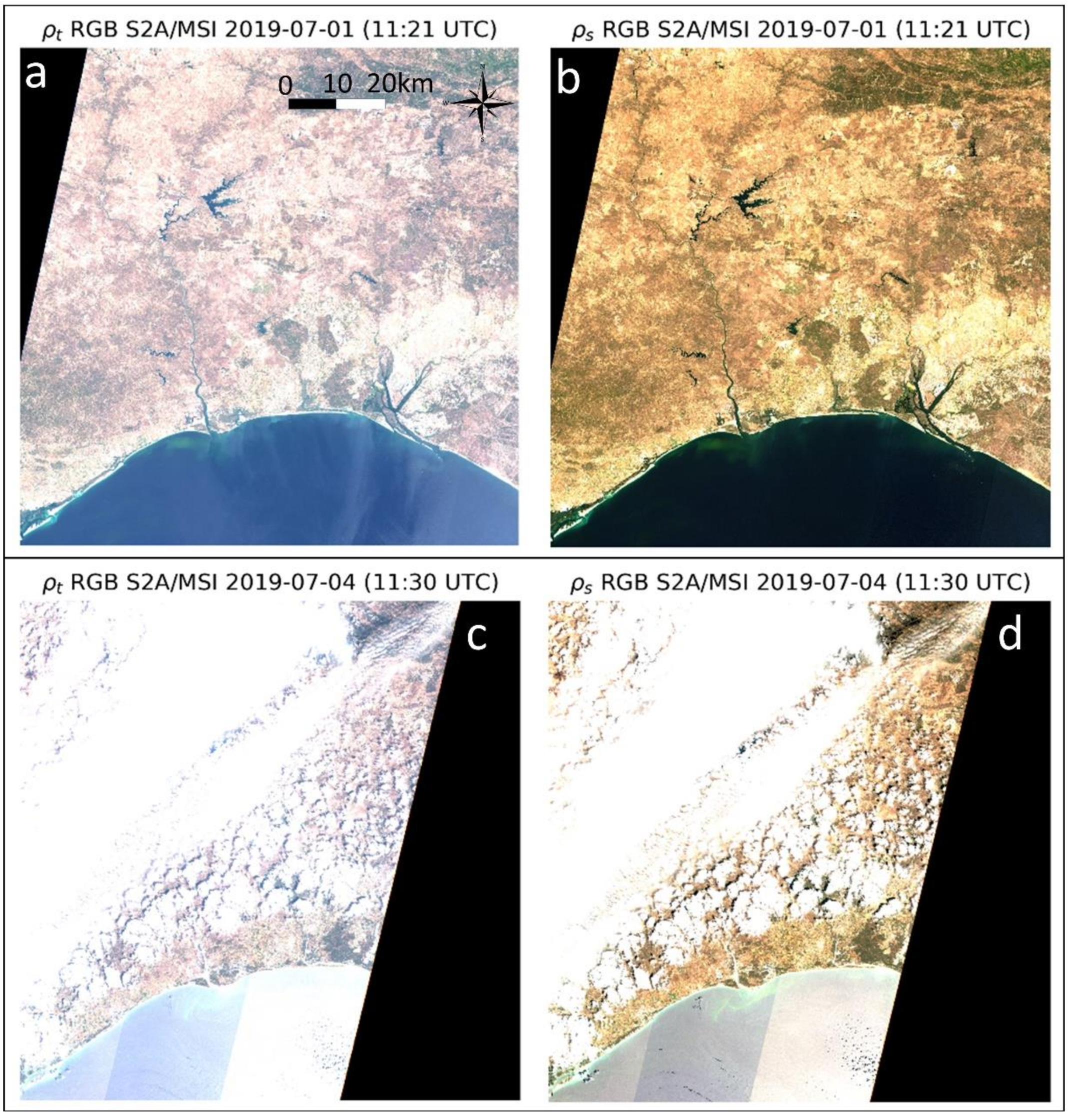

Fig. S1. Examples of the RGB (bands 4-3-2) composites at top of atmosphere-TOA (ρ_t) and bottom of atmosphere-BOA or surface (ρ_s) levels (before and after the ACOLITE atmospheric correction, respectively) for S2 scenes acquired on a, b) 1 and c, d) 4 July 2019 over the Guadiana estuary region. Extreme sun glint effects are clearly observed on 4 July 2019, at both TOA (c) and BOA (d) levels, due to the tile location on the eastern side of the swath, compared with those of 1 July 2019 (a,b), located on the western side of the swath.
